# Control of Far-Field Coupling in Bipartite Nanodisk Arrays

**DOI:** 10.1021/acs.nanolett.6c01863

**Published:** 2026-07-21

**Authors:** Juan J. Alvarez-Serrano, Vincenzo Aglieri, Muhammad Sohaib, Juan R. Deop-Ruano, Andrea Toma, Alejandro Manjavacas

**Affiliations:** † Instituto de Química Física Blas Cabrera (IQF), CSIC, 28006 Madrid, Spain; ‡ 121451 Istituto Italiano di Tecnologia, via Morego 30, 16163 Genova, Italy; ¶ Dipartimento di Fisica, Università degli Studi di Genova, via Dodecaneso 33, 16146 Genova, Italy

**Keywords:** lattice resonances, periodic arrays, nanoparticle arrays, bipartite unit cell, plasmonic crystals, electromagnetic interactions

## Abstract

The optical response of a pair of nanostructures is shaped by the interactions between their localized electromagnetic modes. These are significant only at subwavelength distances and are hence governed by near-field coupling, which limits the range and tunability of emergent phenomena. In contrast, periodic arrays support extended modes known as lattice resonances. When two identical arrays are combined into a bipartite configuration, their lattice resonances interact through far-field coupling, exhibiting nonmonotonic behavior where both the strength and the sign of the interaction depend on their relative positioning. Exploiting this effect, we demonstrate that the optical response of bipartite arrays of silver nanodisks can be precisely engineered by adjusting their relative displacement. To that end, we analyze four different configurations supporting super- and subradiant lattice resonances, noninteracting coexisting modes, and out-of-plane polarized resonances. These findings highlight the rich physics of lattice resonance interactions and offer a versatile approach to designing advanced nanophotonic devices.

## Graphical abstract

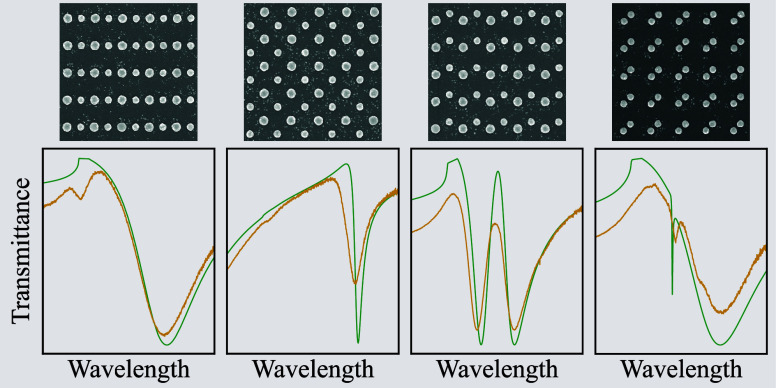

The optical response of a pair of nanostructures is governed by the interaction between their localized electromagnetic modes. In metallic nanostructures, localized surface plasmon resonances couple through near-field interactions, producing optical responses that differ markedly from those of isolated elements.^1–3^ However, these interactions are inherently short-ranged and become significant only at subwavelength separations,^4–6^ limiting the range and tunability of the resulting optical phenomena.^7–9^

In contrast, periodic arrays of metallic nanostructures support collective lattice resonances,^10–14^ which emerge from the multiple scattering among their constituents. These resonances appear at wavelengths commensurate with the period of the array^15–18^ and, due to their collective nature, couple strongly to light,^19–21^ exhibiting exceptionally narrow spectral lineshapes.^22–25^ Such distinctive features are being harnessed in a variety of applications, which range from ultrasensitive biosensing^26–29^ to nanoscale light emission,^30–34^ as well as light-to-heat conversion,^35,36^ chiral light manipulation,^37–39^ energy harvesting,^40–42^ energy transfer,^43,44^ and the realization of strong coupling,^45–47^ to cite some.

When two or more arrays with identical lattices are combined, the resulting multipartite system exhibits an optical response governed by the interaction between their lattice resonances.^48–55^ Unlike the localized modes supported by individual nanostructures, lattice resonances are fully delocalized collective modes that extend across the entire array.^56^ Consequently, their interaction is mediated by the far-field components of the electromagnetic field. This coupling leads to a nonmonotonic behavior, where both the magnitude and sign of the interaction depend on the relative displacement between the arrays,^52^ enabling precise control and paving the way for advanced engineering of collective optical responses.

In this letter, we demonstrate that the optical response of bipartite nanodisk arrays can be precisely controlled through the relative positions of the nanostructures within the unit cell. Building on our previous theoretical studies,^52,53,57^ we fabricate and characterize four representative configurations that illustrate the range of optical behaviors accessible through lattice-resonance coupling. These include superradiant and subradiant configurations, exhibiting enhanced and suppressed radiative losses, respectively, a noninteracting configuration supporting two independent lattice resonances, and a geometry that enables the excitation of an out-of-plane lattice resonance under normal incidence. In all cases, experimental measurements show great agreement with full-wave solutions of Maxwell’s equations obtained via the finite element method (FEM), which further provide insight into the charge distributions and near-field enhancements associated with each lattice resonance. By elucidating the far-field coupling mechanisms in bipartite arrays and translating this understanding into experimentally validated design principles, our results provide a simple and efficient strategy to achieve lattice resonances with tailored functionalities, thereby paving the way for their integration into advanced nanophotonic devices.

Let us consider two nanostructures whose size is much smaller than the wavelength of light, λ, so that they can be modeled as point dipoles **p**_*i*_, each characterized by a polarizability tensor **α**_*i*_. This approximation allows us to use the coupled dipole model (CDM), in which the dipole induced in each element of the pair is given by **p**_*i*_ = **α**_*i*_ **E**_*i*_ + **α**_*i*_ **G**(**R**_*ij*_)**p**_*j*_ with *i* ≠ *j*, where **E**_*i*_ denotes the incident electric field with amplitude *E*_inc_ and **G**(**R**_*ij*_) represents the Green’s tensor of a homogeneous medium, which describes the interaction between two electric dipoles separated by a relative displacement $R_{ij}=\left(x_{i}-x_{j}\right)\mathrm{x̂}+\left(y_{i}-y_{j}\right)ŷ$ . This tensor is defined as $G\left(R_{ij}\right)=\left[k^{2}I_{3\times 3}+n^{-2}\nabla \nabla \right]\;\exp\left(ikn\left|R_{ij}\right|\right)/\left|R_{ij}\right|$ , where $I_{3\times 3}$ is the 3 × 3 identity matrix, *n* is the refractive index of the medium, and *k* = 2π/λ. Solving the CDM equations yields the following induced dipole in each of the two nanostructures:

1 $$\left[\begin{array}{l} p_{1}\\ p_{2} \end{array}\right]={\left[\begin{array}{cc} \alpha_{1}^{-1} & -G\left(R_{12}\right)\\ -G\left(R_{12}\right) & \alpha_{2}^{-1} \end{array}\right]}^{-1}\left[\begin{array}{l} E_{1}\\ E_{2} \end{array}\right]$$

If instead we consider two arrays of nanostructures with the same lattice vectors, the dipole induced in each array satisfies $p_{i}\left(k_{∥}\right)=\alpha_{i}E_{i}\left(k_{∥}\right)+\alpha_{i}G\left(k_{∥},0\right)p_{i}\left(k_{∥}\right)+\alpha_{i}G\left(k_{∥},R_{ij}\right)p_{j}\left(k_{∥}\right)$ , with *i* ≠ *j* (see the Supporting Information), where **k**_∥_ denotes the component of the wavevector parallel to the array and $G\left(k_{∥},R_{ij}\right)$ is the lattice sum.^52^ The latter describes the collective interaction between the nanostructures composing the array and is defined as $G\left(k_{∥},R_{ij}\right)$ = ∑_ν_^′^ **G**(**T**_ν_ + **R**_*ij*_) exp­(−i**k**_∥_·**T**_ν_), where **T**_ν_ stands for the position of the νth unit cell. Importantly, the prime indicates that if *j* = *i*, the term ν = 0 is excluded from the summation to avoid accounting for the interaction of a dipole with itself. Solving for **p**_*i*_(**k**_∥_) yields an expression analogous to eq 1:

2 $$\left[\begin{array}{l} p_{1}\left(k_{∥}\right)\\ p_{2}\left(k_{∥}\right) \end{array}\right]={\left[\begin{array}{cc} A_{1}^{-1}\left(k_{∥}\right) & -G\left(k_{∥},R_{12}\right)\\ -G\left(k_{∥},R_{12}\right) & A_{2}^{-1}\left(k_{∥}\right) \end{array}\right]}^{-1}\left[\begin{array}{l} E_{1}\left(k_{∥}\right)\\ E_{2}\left(k_{∥}\right) \end{array}\right]$$

where $A_{i}^{-1}=\alpha_{i}^{-1}-G\left(k_{∥},0\right)$ .

Comparing eqs 1 and 2 reveals two key insights. First, $A_{i}$ acts as the effective polarizability of the *i*th array, encoding both the response of the individual nanostructures through **α**_*i*_ and their mutual interactions through $G\left(k_{∥},0\right)$ . Second, the term $G\left(k_{∥},R_{12}\right)$ governs the interaction between the two arrays, analogous to the role of **G**(**R**_12_) for a pair of nanostructures. However, the spatial characteristics of these interaction terms differ drastically, as demonstrated in Figure 1a,b. To simplify the analysis, we restrict our discussion to square arrays illuminated at normal incidence (i.e., **k**_∥_ = 0) with an electric field polarized along the *x* axis. Under these conditions, the problem reduces to a scalar formulation involving only the *x* component of the induced dipole and electric field and the *xx* components of the interaction terms.^52^ Accordingly, unless otherwise specified, we omit vector notation and the explicit dependence on **k**_∥_ in all variables in the following discussion.

**Figure 1:**
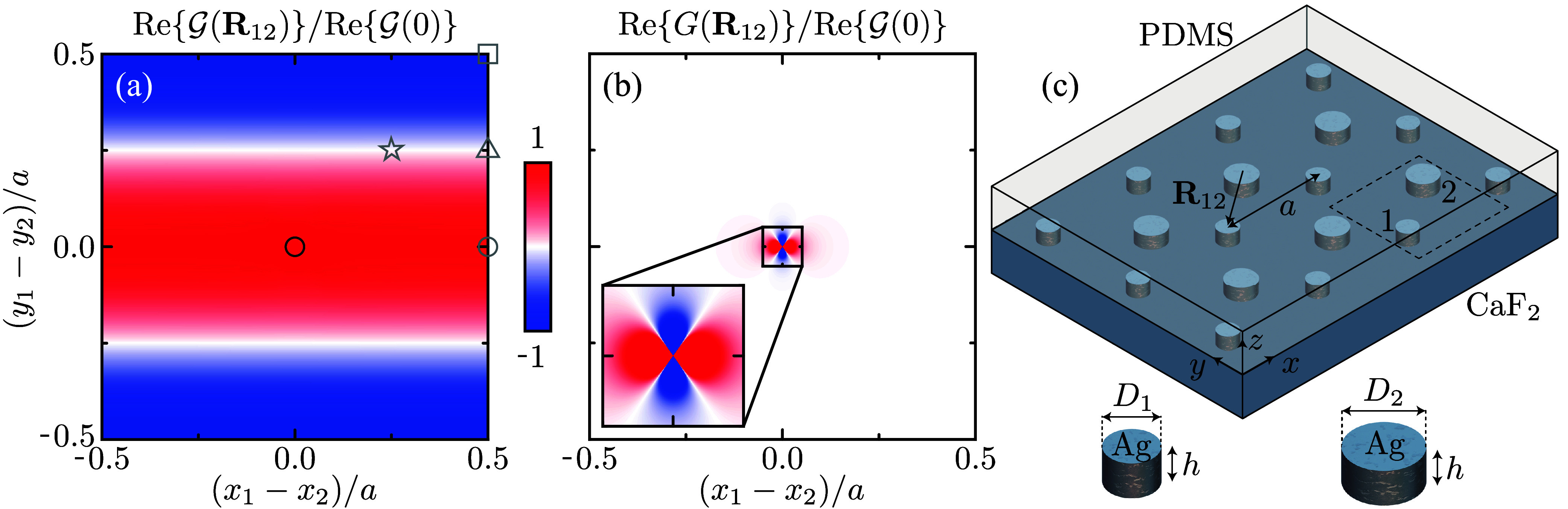
(a) Real part of the *xx* component of the lattice sum, Re{G(R12)} , calculated at **k**_∥_ = 0 for two square arrays of period *a* embedded in a homogeneous medium with refractive index *n*, plotted as a function of their relative displacement. (b) Real part of the *xx* component of the Green’s tensor, Re{G(R12)} , of a homogeneous medium with refractive index *n*, plotted as a function of **R**_12_. We perform all calculations at λ = (1 + 10^–6^)*an*, corresponding to a wavelength marginally exceeding the first Rayleigh anomaly of the arrays, and normalize the results to Re{G(0)} . The inset in (b) shows a magnified view of the region where Re­{*G*(**R**_12_)} takes appreciable values. (c) Schematic view of the system under study, consisting of a bipartite array of silver nanodisks fabricated on a CaF_2_ substrate and coated with a PDMS superstrate. The unit cell, outlined by the dashed black square, contains two nanodisks with height *h* and diameters *D*_1_ and *D*_2_. The relative positions of the nanodisks within the unit cell for the different bipartite configurations considered in this work are indicated schematically in (a). In all cases, the larger nanodisk is located at the origin (black circle), while the smaller nanodisk occupies different positions depending on the configuration, as denoted by the gray markers: superradiant (circle), subradiant (square), noninteracting (triangle), and out-of-plane (star).

Figure 1a shows the real part of the *xx* component of the lattice sum, $Re\left\{G\left(R_{12}\right)\right\}$ , as a function of the relative displacement between two square arrays with period *a*, embedded in a medium with refractive index *n*. We perform the calculation at a wavelength λ = (1 + 10^–6^)*an*, slightly above the spectral position of the first Rayleigh anomaly of the arrays, and normalize the results to Re{G(0)} . The results reveal that $Re\left\{G\left(R_{12}\right)\right\}$ inherits the symmetries of both the square lattice and the *x*-polarized driving field. Inversion symmetry requires it to be even in both (*x*_1_ – *x*_2_) and (*y*_1_ – *y*_2_). More importantly, polarization along *x* breaks the fourfold rotational symmetry of the square lattice down to a twofold one. This accounts for the striking anisotropy visible in the figure: since the far-field radiation pattern of an *x*-polarized dipole vanishes along *x* and peaks along *y*, the coupling is nearly independent of displacement along *x* but varies strongly along *y*. The sign changes of $Re\left\{G\left(R_{12}\right)\right\}$ with increasing |*y*_1_ – *y*_2_|/*a* further reflect the oscillatory nature of this radiation pattern. Figure 1b presents the corresponding analysis for the *xx* component of the Green’s tensor, Re­{*G*(**R**_12_)}. We consider only the real parts because the imaginary components are negligible, as confirmed in Figure S2.

Upon examination of Figure 1a,b, it is evident that $Re\left\{G\left(R_{12}\right)\right\}$ varies continuously between −Re{G(0)} and Re{G(0)} across the entire range of possible relative displacements. Consequently, its value can be smoothly tuned, and even completely nullified, by adjusting the arrangement of nanostructures within the unit cell of the bipartite array. In sharp contrast, Re­{*G*(**R**_12_)} remains appreciable only at separations significantly smaller than λ/*n* (see inset). Accordingly, although a sign change is also observed in the region considered, these variations are restricted to deeply subwavelength distances and are effectively inaccessible experimentally owing to the finite dimensions of the nanostructures.

The analysis of $Re\{G\left(R_{12}\right)\}$ , which forms the basis of the hybridization approach introduced in our previous work,^52^ clearly demonstrates that the interaction between collective lattice resonances supported by two periodic arrays is considerably richer and more versatile than that between localized modes of individual nanostructures, enabling effective modulation of both the magnitude and the sign of the coupling.

Building on this result, we designed, fabricated, and characterized four bipartite arrays for which $Re\left\{G\left(R_{12}\right)\right\}$ takes distinct values, leading to markedly different optical responses (see Figure 1a). These examples illustrate how the optical behavior of bipartite arrays can be tailored through coupling between lattice resonances. Figure 1c presents a schematic of the arrays under consideration. Each system is formed by combining two square arrays with period *a* = 550 nm, laterally displaced with respect to each other by **R**_12_. The arrays consist of silver nanodisks with height *h* = 90 nm and diameters *D*_1_ and *D*_2_. They are fabricated via electron-beam lithography on a CaF_2_ substrate, subsequently encapsulated in PDMS, and optically characterized using the microspectroscopic setup described in the Supporting Information. To calculate their optical response, we solve Maxwell’s equations using the FEM, employing tabulated dielectric functions for silver, CaF_2_, and PDMS, taken respectively from refs 58, 59, and 60.

We begin by analyzing the behavior of two monopartite arrays, which serve as building blocks for the bipartite configurations discussed later. Figure 2a shows the transmittance spectra of two arrays of silver nanodisks with diameters *D*_1_ = 140 nm (green curves) and *D*_2_ = 180 nm (orange curves). Solid curves correspond to experimental measurements, which exhibit very good agreement with the FEM calculations shown by the dashed curves. The insets display scanning electron microscopy (SEM) images of the fabricated samples and a schematic representation of the unit cell for each array. Both monopartite arrays support a lattice resonance, with the one sustained by the array of smaller nanodisks appearing closer to the Rayleigh anomaly and exhibiting a higher quality factor. These are signatures of a more collective character, as demonstrated in ref 61, and therefore this resonance is expected to be more sensitive to fabrication imperfections, such as the truncated conical shape of the nanodisks, surface roughness, grain boundaries, and the formation of a native oxide layer. The enhanced sensitivity of this lattice resonance explains why its measured transmittance dip deviates more from the FEM calculations than that of the resonance of the other array.^61^

**Figure 2:**
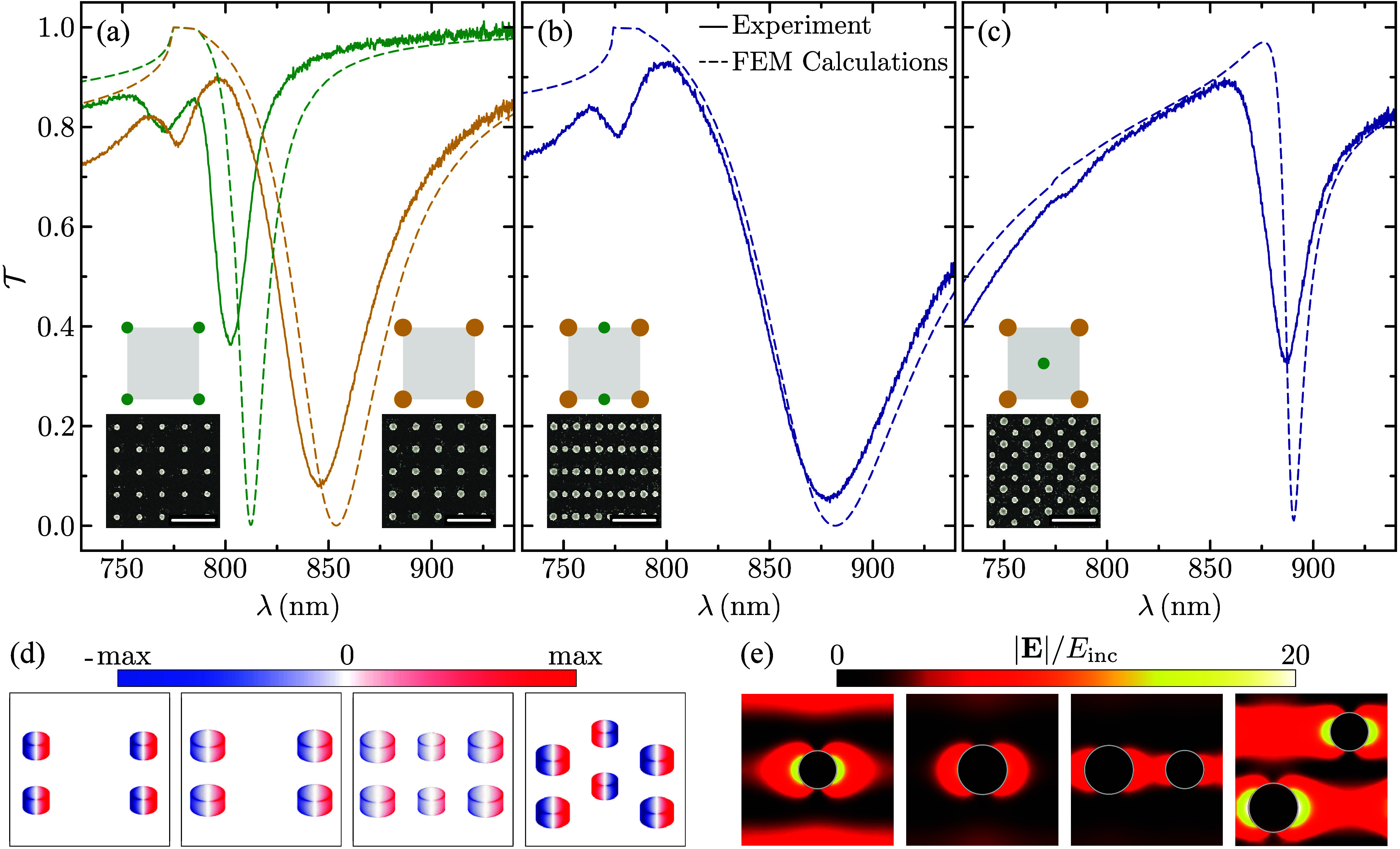
(a) Transmittance spectra for two monopartite arrays composed of nanodisks with diameters *D*_1_ = 140 nm (green curves) and *D*_2_ = 180 nm (orange curves). (b, c) Transmittance spectra for bipartite arrays in the superradiant (b) and subradiant (c) configurations, built from the combination of the two monopartite arrays in (a). In all cases, solid and dashed curves represent experimental measurements and FEM calculations, respectively. Each inset shows a schematic of the nanodisk arrangement within the unit cell along with an SEM image of the fabricated array (the scale bars represent 1 μm). (d) Charge density on the surface of the nanodisks. (e) Electric field enhancement across the unit cell, evaluated at the plane *z* = *h*/2. Both the charges and field enhancements were calculated at the wavelength of the corresponding lattice resonance.

These results indicate that tuning lattice resonances in monopartite arrays requires significant modifications of the individual constituents, particularly their size, since the collective response is strongly tied to their intrinsic properties. Even so, the resonance strength, spectral position, and quality factor remain interdependent, making precise control highly challenging. In contrast, leveraging the interaction between lattice resonances in bipartite arrays provides a far more versatile approach, enabling greater flexibility in tuning optical responses. To demonstrate this, we examine two bipartite configurations that maximize the interaction strength, introduced in our previous theoretical works.^52,53^ Specifically, we consider unit cells where the relative displacement between nanostructures is $R_{12}=\mathrm{x̂}a/2$ and $R_{12}=\left(\mathrm{x̂}+ŷ\right)a/2$ . For these configurations, $Re\left\{G\left(R_{12}\right)\right\}$ is equal to Re{G(0)} in the first case, whereas in the second case it becomes −Re{G(0)} , as illustrated in Figure 1a.

Mathematically, the lattice resonances supported by these arrays correspond to the poles of the matrix that connects the induced dipoles to the external field, as defined in eq 2. The pole condition reads α_1_^–1^α_2_^–1^ – (α_1_^–1^ + α_2_^–1^)­ G(0) + ${\left[G\left(0\right)\right]}^{2}$ – ${\left[G\left(R_{12}\right)\right]}^{2}$ = 0. For the bipartite arrays considered here, for which, neglecting the imaginary parts (see Figure S2), $G\left(R_{12}\right)$ ≈ ±G(0) , this condition reduces to α_1_^–1^α_2_^–1^/(α_1_^–1^ + α_2_^–1^) ≈ G(0) . From eq 2, the dipoles induced at the lattice resonance satisfy

$$\frac{p_{1}}{p_{2}}=\frac{\alpha_{2}^{-1}-G\left(0\right)+G\left(R_{12}\right)}{\alpha_{1}^{-1}-G\left(0\right)+G\left(R_{12}\right)}$$

In the first bipartite configuration, where $G\left(R_{12}\right)\approx G\left(0\right)$ , we find that the dipole ratio is *p*_1_/*p*_2_ ≈ α_1_/α_2_. Since the lattice resonance occurs at wavelengths well above those of the localized plasmons, this ratio is positive, indicating in-phase oscillation of the dipoles within the unit cell. The resulting enhancement of far-field radiation gives rise to a superradiant lattice resonance with high reflectance, low absorbance, and a broad linewidth.

In contrast, for the second bipartite array, where the interaction term satisfies $G\left(R_{12}\right)\approx -G\left(0\right)$ , the induced dipoles obey *p*_1_/*p*_2_ ≈ −α_1_/α_2_. This indicates antiphase dipole oscillations at the lattice resonance. The associated reduction of far-field radiation yields a subradiant resonance with lower reflectance, higher absorbance, and a narrower linewidth than its superradiant counterpart.

All these predictions, based on the CDM, are validated by the measured transmittance spectra shown in Figure 2b,c. As in the case of the monopartite arrays, the measurements exhibit very good agreement with the corresponding FEM calculations. The superradiant configuration displays a broad lattice resonance with a quality factor of *Q* ≈ 9 (from the calculated spectra), markedly lower than those of the two monopartite arrays (*Q* ≈ 48 for *D*_1_ = 140 nm and *Q* ≈ 16 for *D*_2_ = 180 nm). In contrast, the subradiant configuration supports a significantly narrower resonance with *Q* ≈ 64. The calculated reflectance and absorbance spectra, shown in Figure S3c,d, corroborate these predictions: the superradiant resonance exhibits higher peak reflectance (0.98 versus 0.82), while the subradiant resonance shows an order-of-magnitude larger absorbance (0.20 versus 0.02).

The charge densities displayed in Figure 2d further confirm the super- and subradiant nature of these lattice resonances. Their dipolar character validates the dipolar approximation and agrees with our predictions: the dipoles oscillate in phase in the superradiant configuration and in antiphase in the subradiant configuration. Consistently, the near-field maps in Figure 2e show a weaker field enhancement for the superradiant mode and a stronger enhancement for the subradiant mode, reflecting their efficient radiation and field confinement, respectively.

The results of Figure 2 demonstrate that the optical response of bipartite arrays can be strongly modified through the relative displacement of the nanostructures within the unit cell. Those configurations maximize the interaction; however, as shown in Figure 1a, there also exist displacements for which the coupling vanishes, i.e., $Re\left\{G\left(R_{12}\right)\right\}=0$ . Under these conditions, eq 2 predicts that the bipartite response reduces to the superposition of the responses of the two monopartite arrays. To explore this scenario, Figure 3a presents the transmittance spectra for a bipartite array with **R**_12_ = (**x̂**/2 + **ŷ**/4)*a*. Solid and dashed curves represent experimental measurements and FEM calculations, respectively, showing again very good agreement. As anticipated, the spectra exhibit two distinct lattice resonances. When compared with the calculated transmittance spectra of the monopartite arrays, shown as a shaded background, the resonance at shorter wavelengths (λ ≈ 816 nm) can be attributed to the monopartite array of nanodisks with diameter *D*_1_ = 140 nm (green), whereas the resonance at longer wavelengths (λ ≈ 857 nm) corresponds to the array with *D*_2_ = 180 nm (orange).

**Figure 3:**
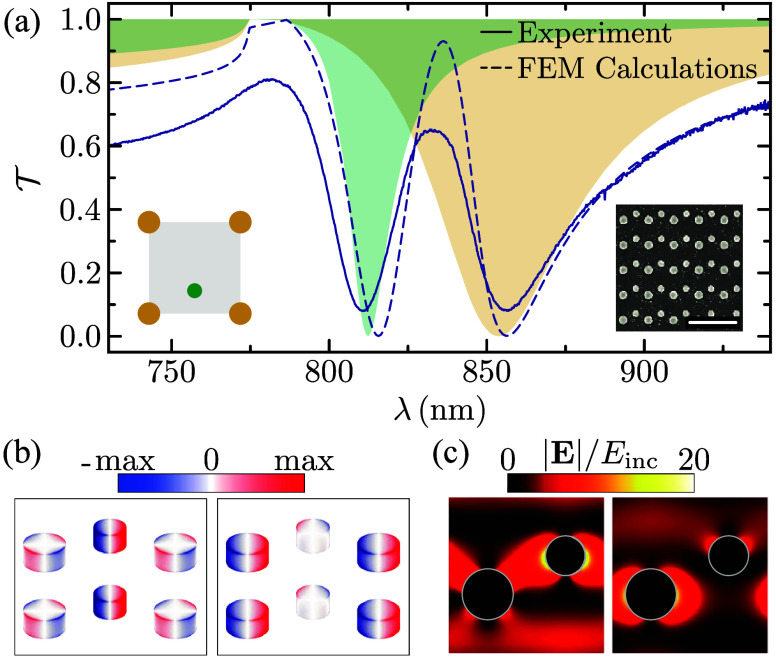
(a) Transmittance spectra for a bipartite array of nanodisks in the noninteracting configuration. Solid and dashed curves represent experimental measurements and FEM calculations, respectively. The insets show a schematic of the nanodisk arrangement within the unit cell and an SEM image of the fabricated array (the scale bar represents 1 μm). For reference, the shaded areas indicate the calculated transmittance spectra of the monopartite arrays of nanodisks with diameters *D*_1_ = 140 nm (green) and *D*_2_ = 180 nm (orange). (b) Charge density on the surface of the nanodisks. (c) Electric field enhancement across the unit cell, evaluated at the plane *z* = *h*/2. Both the charges and field enhancements are calculated at the wavelengths of the two different lattice resonances.

The charge densities shown in Figure 3b further support this interpretation. For the shorter-wavelength resonance, the smaller nanodisk exhibits a dipolar pattern, while the larger one shows a weak quadrupolar response, indicating a residual higher-order coupling. The roles reverse for the longer wavelength resonance. The corresponding near-field maps, shown in Figure 3c, explain the suppression of the dipolar coupling: the resonant nanodisk is located at a field maximum, while the other lies in a region where the field vanishes and is effectively decoupled from the resonance. The finite size of the nanodisks, not captured by the pointlike nature of the dipoles used in the CDM, gives rise to weak residual coupling, consistent with the observed quadrupolar charge distributions.

Although our focus is on normal incidence, the behavior of all three configurations is preserved under oblique illumination. As shown in Figures S4 and S5, increasing the incidence angle redshifts and narrows the lattice resonances of the superradiant and subradiant arrays, consistent with the shift of the Rayleigh anomaly. In the noninteracting configuration, the longer wavelength resonance behaves similarly, whereas the shorter wavelength resonance remains nearly unchanged.

The optical responses discussed so far are dominated by interactions between electric dipoles. Metallic nanostructures, however, also support magnetic dipoles which, under suitable conditions, can couple to the electric dipoles and give rise to unique optical phenomena. One example is the out-of-plane lattice resonance emerging at normal incidence, recently predicted in ref 57. This mode is sustained by a bipartite array in which **R**_12_ = (**x̂**/4 + **ŷ**/4)*a*. The array is excited at normal incidence with electric and magnetic fields polarized along **ŷ**′ = (**x̂** + **ŷ**)/ $\sqrt{2}$ and **x̂**′ = (**x̂** − **ŷ**)/ $\sqrt{2}$ , respectively. For this arrangement, as shown in Figure S6, all the interactions among the different components of the electric dipoles cancel out. In contrast, the interaction between the out-of-plane electric dipoles (along the *z* axis) and the in-plane magnetic dipoles (along the *x*′ axis), and vice versa, is maximized.

Following the derivation in the Supporting Information, the out-of-plane electric dipoles satisfy *p*_*i*,*z*_ ≈ α_*i*,*z*_^EE^ $[G_{zz}^{EE}\left(0\right)p_{i,z}$ + $G_{zx^{\prime }}^{EM}\left(R_{ij}\right)m_{j,x^{\prime }}]$ , while the in-plane magnetic dipoles are given by $m_{j,x^{\prime }}\approx \alpha_{j,x^{\prime }}^{MM}H_{inc}$ , where *H*_inc_ is the amplitude of the incident magnetic field. Solving these relations leads to *p*_*i*,*z*_ ≈ $A_{i,zz}^{EE}G_{zx^{\prime }}^{EM}\left(R_{ij}\right)\alpha_{j,x^{\prime }}^{MM}H_{inc}$ . This means that the out-of-plane lattice resonance supported by the electric dipoles, which is encoded in $A_{i,zz}^{EE}$ , can be excited through the in-plane magnetic dipoles induced by the incident field via the electric-magnetic interaction $G_{zx^{\prime }}^{EM}\left(R_{ij}\right)$ . Furthermore, at the lattice resonance, *p*_1,*z*_/*p*_2,*z*_ = $G_{zx^{\prime }}^{EM}\left(R_{12}\right)/G_{zx^{\prime }}^{EM}\left(R_{21}\right)$ = −1, indicating antiphase oscillation of the out-of-plane dipoles.

To verify these predictions, we fabricated and characterized a bipartite array of identical silver nanodisks (*D* = 160 nm) with the unit-cell configuration described above. The height and period remain fixed at *h* = 90 nm and *a* = 550 nm. The experimental transmittance spectrum is shown in Figure 4a (solid curve) and exhibits very good agreement with the corresponding FEM calculations (dashed curve). Both spectra display two distinct dips. The broader dip at longer wavelengths corresponds to an in-plane lattice resonance (see Figure S7), whereas the narrower dip at shorter wavelengths is the predicted out-of-plane mode. The latter is confirmed by the charge density in Figure 4b, which reveals out-of-plane dipoles oscillating in antiphase. This configuration enhances the subradiant character of the resonance, resulting in a high quality factor (*Q* ≈ 390 for the calculated spectrum) and strong absorbance (see Figure S3f). It also produces strong electric and magnetic field enhancement within the unit cell (Figure 4c,d), with the magnetic field exceeding 60 times the incident amplitude in the gap between the nanodisks.

**Figure 4:**
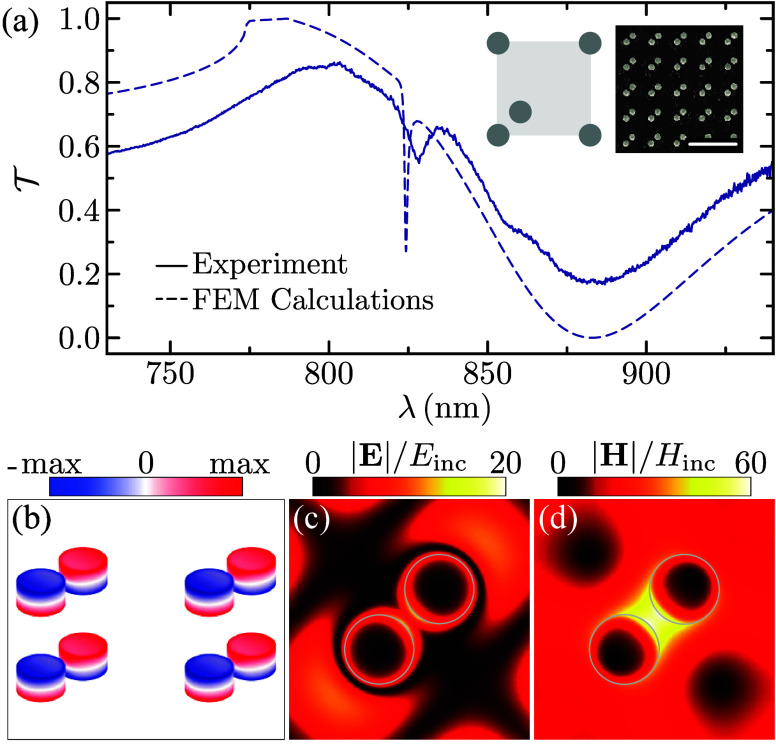
(a) Transmittance spectra for a bipartite array of nanodisks in the out-of-plane configuration. The two nanodisks in the unit cell are identical, with diameter *D* = 160 nm. Solid and dashed curves represent experimental measurements and FEM calculations, respectively. The insets show a schematic of the nanodisk arrangement within the unit cell and an SEM image of the fabricated array (the scale bar represents 1 μm). (b) Charge density on the surface of the nanodisks. (c) Electric and (d) magnetic field enhancements across the unit cell, evaluated at the plane *z* = *h*/2. Both the charges and field enhancements are calculated at the wavelength of the out-of-plane lattice resonance (λ = 824.3 nm).

Although the main appeal of the out-of-plane mode lies in its excitation at normal incidence, for completeness, we show in Figure S8 its evolution under oblique incidence. As in the other configurations, the lattice resonance redshifts with increasing angle of incidence. Interestingly, an additional resonance emerges in the spectrum, exhibiting characteristics consistent with those of a bound state in the continuum.

In summary, we have demonstrated that the optical response of a bipartite array is governed by, and can be engineered through, the interaction between the lattice resonances of its two underlying monopartite arrays. Using a coupled-dipole framework, we have shown that this interaction is controlled by the lattice sum, whose magnitude and sign can be tuned through the relative displacement of the nanostructures within the unit cell. We have validated these predictions by fabricating and characterizing different arrays, obtaining experimental results in great agreement with full-wave solutions of Maxwell’s equations. In particular, we have realized configurations supporting superradiant and subradiant lattice resonances, characterized respectively by enhanced and suppressed radiative coupling, as well as a noninteracting configuration in which the response reduces to the superposition of the lattice resonances of the individual monopartite arrays. We have further demonstrated a bipartite geometry that enables the excitation of an out-of-plane lattice resonance under normal-incidence illumination through electric-magnetic coupling. The physical understanding of the far-field coupling mechanism developed in this work, together with the general design principles derived from it and their high-fidelity experimental validation, establishes bipartite arrays as a powerful and versatile platform for engineering lattice resonances with unprecedented control over radiative coupling, near-field enhancement, and polarization. These results open new pathways for the development of high-performance photonic systems beyond what is achievable with conventional monopartite architectures.

## Supplementary Material



## Notes

Description of the fabrication and optical characterization of the arrays, description of the FEM calculations and the coupled dipole model, and Figures S1–S8 (PDF)
