# Supplementary material for: Control of Far-Field Coupling in Bipartite Nanodisk Arrays
Source: Nano Lett. 2026 Jul 21;26(30):9821–8. doi: 10.1021/acs.nanolett.6c01863 (PMC13450425; doi:10.1021/acs.nanolett.6c01863)
Supplement: Supplementary file 1 [file nl6c01863_si_001.pdf]

# Supporting Information: Control of Far-Field Coupling in Bipartite Nanodisk Arrays

Juan J. Alvarez-Serrano [†,§] Vincenzo Aglieri [‡,§] Muhammad Sohaib [‡,¶] Juan R. Deop-Ruano [†] Andrea Toma [*,‡] and Alejandro Manjavacas [*,†]

†*Instituto de Química Física Blas Cabrera (IQF), CSIC, 28006 Madrid, Spain*

‡*Istituto Italiano di Tecnologia, via Morego 30, 16163 Genova, Italy*

¶*Dipartimento di Fisica, Università degli Studi di Genova, via Dodecaneso 33, 16146 Genova, Italy*

§*These authors contributed equally to this work.*

E-mail: andrea.toma@iit.it; a.manjavacas@csic.es

## Table of Contents

- Fabrication of the Bipartite Arrays .......... S-2
- Microspectroscopic Optical Characterization .......... S-2
- Full-Wave Solutions of Maxwell's Equations .......... S-3
- Coupled Dipole Model for Bipartite Arrays .......... S-4
- Coupled Dipole Model for a Bipartite Array in the Out-of-Plane Configuration .......... S-5
- Figure S2 .......... S-7
- Figure S3 .......... S-8
- Figure S4 .......... S-9

- Figure S5 .......... S-10
- Figure S6 .......... S-11
- Figure S7 .......... S-12
- Figure S8 .......... S-13


## Fabrication of the Bipartite Arrays

The periodic arrays of nanodisk were fabricated on calcium fluoride (100) substrates cleaned in a three-steps ultrasonic bath of acetone, isopropyl alcohol (IPA) and deionized water (1 minute at 37 kHz frequency), followed by an oxygen plasma ashing (3 minutes, RF power 100 W). The cleaned substrates were spin coated (1 minute at 1800 rpm) with $\sim 3\%$ solution of polymethyl methacrylate (PMMA 950k) electron resist diluted in anisole. Then, the 120 nm thick PMMA layer was thermally covered with a 12 nm thick sacrificial aluminum (Al) layer to avoid charging effects during electron beam lithography (EBL) process, operated by a RAITH 150-Two system (20 kV accelerating voltage, 20 $\mu$m aperture and exposure dose of 600 $\mu$C/cm$^2$). After the electron beam exposure, the Al layer was removed in a 1 M solution of potassium hydroxide (KOH:$H_2O$), for 20 seconds and stopped in water. The PMMA was developed in a solution of methyl isobutyl ketone and IPA, in a 1:3 ratio for 30 seconds and stopped in IPA for 30 seconds. On the developed PMMA layer, a silver (Ag) 90 nm thick layer was deposited by electron-beam physical vapor deposition, and the Ag excess was lifted off in ultrasonic bath of hot acetone (for 1 minute at 37 kHz frequency). The sample was finally embedded in a thick layer of polydimethylsiloxane (PDMS) to obtain a homogeneous refractive index.

## Microspectroscopic Optical Characterization

The periodic arrays of nanodisks were characterized using the custom-made optical setup in transmission mode shown in Figure S1. Wide spectrum incoherent light generated from a tungsten-halogen lamp (Spectral Products ASBN-W100F-L) was collimated using a 4×

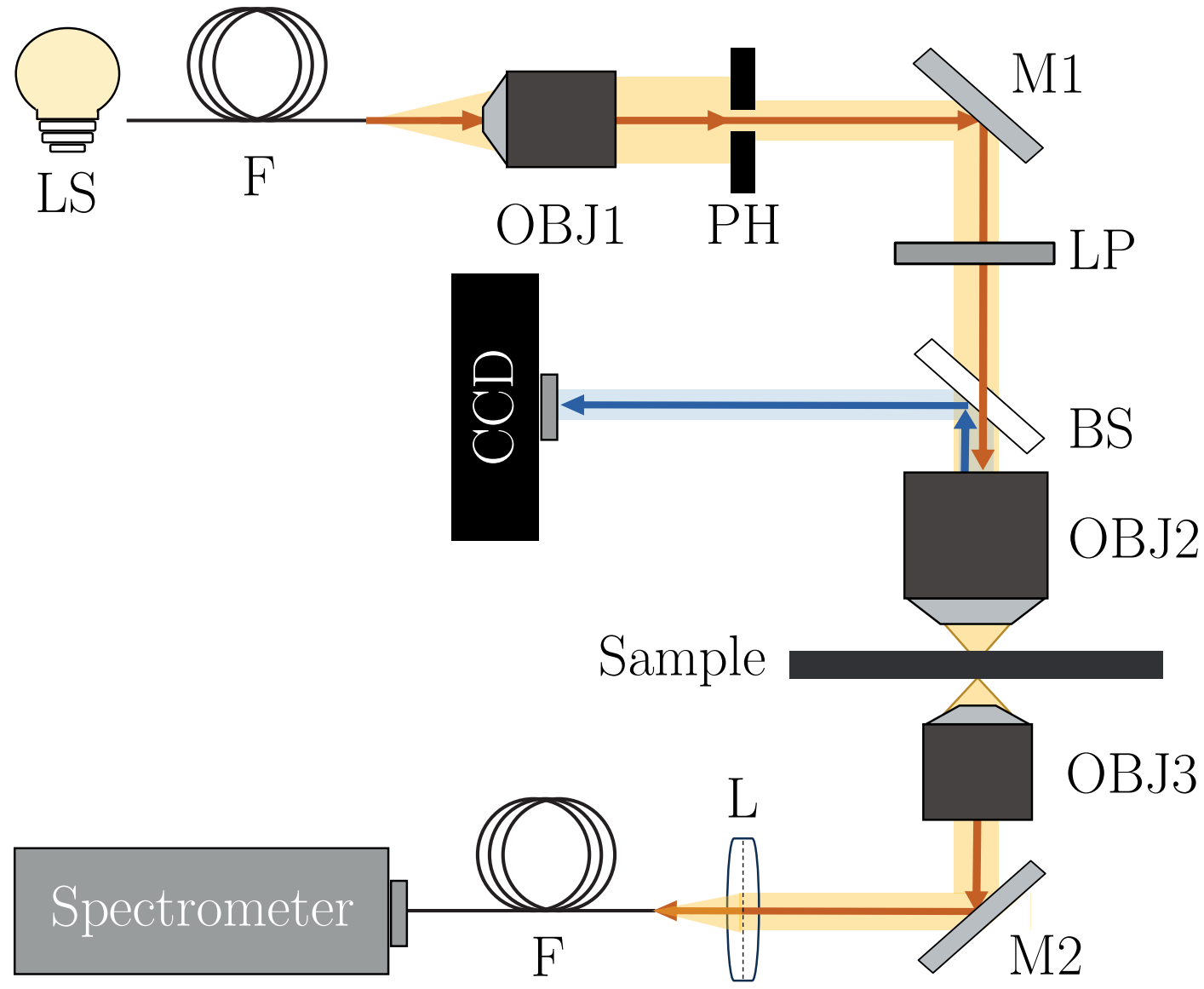


Figure S1: Schematic representation of the custom-made optical setup. LS: incoherent white light emitting lamp; F: fiber cable; L: lens; PH: pinhole; M: mirror; BS: beam splitter; LP: linear polarizer; OBJ: objective lens (OBJ1 = 4× NA 0.1, OBJ2 = 10× NA 0.3, OBJ3 = 4× NA 0.1).

objective lens (Nikon Plan N 4×/NA 0.1) and spatially filtered through a 500 $\mu$m (diameter) pinhole. The light beam was then linearly polarized and focused onto the arrays using a 10× objective lens (Nikon Plan Fluor 10×/NA 0.3), thus obtaining a beam spot size of around 50 $\mu$m on the sample plane. The transmitted signal was collected through a second 4× objective lens and sent into a fiber coupled spectrometer (Ocean Optics USB4000) using a condenser lens. A CCD live imaging camera was employed to easily navigate across the sample surface.

## Full-Wave Solutions of Maxwell's Equations

Full-wave solutions of Maxwell's equations were obtained using the finite element method (FEM), as implemented in the commercial software COMSOL Multiphysics. Owing to the periodicity of the system, it was sufficient to model a single unit cell with periodic boundary conditions. When possible, we exploited the symmetry of the unit cell to reduce the size of

the simulation domain by applying perfect electric conductor and perfect magnetic conductor boundary conditions. Along the direction parallel to the $z$ axis, the computational region was truncated using perfectly matched layers to emulate an open domain. All calculations were verified for convergence with respect to both mesh density and computational domain size.

## Coupled Dipole Model for Bipartite Arrays

We consider a square bipartite array, constructed by combining two monopartite arrays with the same lattice vectors. The dipole induced in the $i$th nanostructure of the $\mu$th unit cell of the array is given by

$$\mathbf{p}_{i,\mu} = \boldsymbol{\alpha}_i \mathbf{E}_{i,\mu} + \boldsymbol{\alpha}_i \sum_{\nu \neq \mu} \mathbf{G}(\mathbf{T}_\mu - \mathbf{T}_\nu)\mathbf{p}_{i,\nu} + \boldsymbol{\alpha}_i \sum_{\nu} \mathbf{G}(\mathbf{R}_{ij} + \mathbf{T}_\mu - \mathbf{T}_\nu)\mathbf{p}_{j,\nu}. \quad \text{(S1)}$$

Here, Latin indices label the nanostructures within a unit cell, while Greek indices indicate the unit cells in the bipartite array. Exploiting the periodicity of the array, we write $\mathbf{p}_{i,\mu} = \mathbf{p}_i(\mathbf{k}_\parallel)\exp(\mathrm{i}\mathbf{k}_\parallel \cdot \mathbf{T}_\mu)$ and $\mathbf{E}_{i,\mu} = \mathbf{E}_i(\mathbf{k}_\parallel)\exp(\mathrm{i}\mathbf{k}_\parallel \cdot \mathbf{T}_\mu)$, where $\mathbf{k}_\parallel$ is the component of the wavevector parallel to the array. Therefore, substituting these expressions into Equation (S1), we obtain

$$\mathbf{p}_i(\mathbf{k}_\parallel) = \boldsymbol{\alpha}_i \left[\mathbf{E}_i(\mathbf{k}_\parallel) + \boldsymbol{\mathcal{G}}(\mathbf{k}_\parallel, 0)\mathbf{p}_i(\mathbf{k}_\parallel) + \boldsymbol{\mathcal{G}}(\mathbf{k}_\parallel, \mathbf{R}_{ij})\mathbf{p}_j(\mathbf{k}_\parallel)\right],$$

where $\boldsymbol{\mathcal{G}}(\mathbf{k}_\parallel, \mathbf{R}_{ij})$ is the lattice sum defined in the main text. The expression above can be rewritten in matrix form as

$$\begin{bmatrix} \boldsymbol{\mathcal{A}}_1^{-1}(\mathbf{k}_\parallel) & -\boldsymbol{\mathcal{G}}(\mathbf{k}_\parallel, \mathbf{R}_{12}) \\ -\boldsymbol{\mathcal{G}}(\mathbf{k}_\parallel, \mathbf{R}_{12}) & \boldsymbol{\mathcal{A}}_2^{-1}(\mathbf{k}_\parallel) \end{bmatrix} \begin{bmatrix} \mathbf{p}_1(\mathbf{k}_\parallel) \\ \mathbf{p}_2(\mathbf{k}_\parallel) \end{bmatrix} = \begin{bmatrix} \mathbf{E}_1(\mathbf{k}_\parallel) \\ \mathbf{E}_2(\mathbf{k}_\parallel) \end{bmatrix},$$

where $\boldsymbol{\mathcal{A}}_i^{-1} = \boldsymbol{\alpha}_i^{-1} - \boldsymbol{\mathcal{G}}(\mathbf{k}_\parallel, 0)$. Solving for the induced dipoles, we arrive to Equation (2) of the main paper.

## Coupled Dipole Model for a Bipartite Array in the Out-of-Plane Configuration

To characterize the bipartite array in the out-of-plane configuration analyzed in Figure 4 of the main paper, we need to consider both electric and magnetic dipoles and their mutual interactions. Assuming normal incidence (i.e. $\mathbf{k}_\parallel = 0$) and dropping the dependence on $\mathbf{k}_\parallel$ for simplicity and consistency with the main text, the electric dipole induced in the $i$th nanostructure of the unit cell and the magnetic dipole induced in the $j$th nanostructure of the same unit cell (with $j \neq i$) approximately satisfy

$$\mathbf{p}_i \approx \boldsymbol{\alpha}_i^{\rm EE} \left[\mathbf{E}_i + \boldsymbol{\mathcal{G}}^{\rm EE}(0)\mathbf{p}_i + \boldsymbol{\mathcal{G}}^{\rm EM}(\mathbf{R}_{ij})\mathbf{m}_j\right],$$
$$\mathbf{m}_j \approx \boldsymbol{\alpha}_j^{\rm MM} \left[\mathbf{H}_j + \boldsymbol{\mathcal{G}}^{\rm MM}(0)\mathbf{m}_j + \boldsymbol{\mathcal{G}}^{\rm ME}(\mathbf{R}_{ji})\mathbf{p}_i\right].$$

Here, the superscripts EE, MM, ME and EM denote the electric-electric, magnetic-magnetic, magnetic-electric, and electric-magnetic contributions, respectively. This expression is obtained by neglecting the terms $\boldsymbol{\mathcal{G}}^{\rm EE}(\mathbf{R}_{ij})$, $\boldsymbol{\mathcal{G}}^{\rm MM}(\mathbf{R}_{ji})$, $\boldsymbol{\mathcal{G}}^{\rm EM}(0)$, and $\boldsymbol{\mathcal{G}}^{\rm ME}(0)$, which is justified by the results shown in Figure S6. Notice that $\boldsymbol{\mathcal{G}}^{\rm MM}(\mathbf{R}_{ij}) = \boldsymbol{\mathcal{G}}^{\rm EE}(\mathbf{R}_{ij})$ and $\boldsymbol{\mathcal{G}}^{\rm EE}(\mathbf{R}_{ij}) = \sum_\nu' \mathbf{G}^{\rm EE}(\mathbf{R}_{ij} + \mathbf{T}_\nu)$, while $\boldsymbol{\mathcal{G}}^{\rm EM}(\mathbf{R}_{ij}) = -\boldsymbol{\mathcal{G}}^{\rm ME}(\mathbf{R}_{ij})/n^2$ and $\boldsymbol{\mathcal{G}}^{\rm ME}(\mathbf{R}_{ij}) = \sum_\nu' \mathbf{G}^{\rm ME}(\mathbf{R}_{ij} + \mathbf{T}_\nu)$. In these expressions, $\mathbf{G}^{\rm EE}(\mathbf{R}_{ij}) = [k^2\mathcal{I}_{3\times3} + n^{-2}\nabla\nabla] \exp({\rm i}kn|\mathbf{R}_{ij}|)/|\mathbf{R}_{ij}|$ and $\mathbf{G}^{\rm ME}(\mathbf{R}_{ij}) = -ik\boldsymbol{\nabla} \times \exp({\rm i}kn|\mathbf{R}_{ij}|)/|\mathbf{R}_{ij}|$.

As explained in the main text, we excite the bipartite array at normal incidence with a plane wave impinging from the top, whose electric and magnetic components are parallel to $\hat{\mathbf{y}}' = (\hat{\mathbf{x}} + \hat{\mathbf{y}})/\sqrt{2}$ and $\hat{\mathbf{x}}' = (\hat{\mathbf{x}} - \hat{\mathbf{y}})/\sqrt{2}$, respectively. Working in the rotated coordinated system $x'y'z$ (see Figure S6), the out-of-plane component of the induced electric dipole and

the in-plane component of the magnetic dipole are approximately given by

$$p_{i,z} \approx \alpha_{i,z}^{\mathrm{EE}} \left[ \mathcal{G}_{zz}^{\mathrm{EE}}(0) p_{i,z} + \mathcal{G}_{zx'}^{\mathrm{EM}}(\mathbf{R}_{ij}) m_{j,x'} \right] ,$$

$$m_{j,x'} \approx \alpha_{j,x'}^{\mathrm{MM}} \left[ H_{\mathrm{inc}} + \mathcal{G}_{x'x'}^{\mathrm{MM}}(0) m_{j,x'} \right] ,$$

where we have neglected the back action of $p_{i,z}$ on $m_{j,x'}$. In these expressions, $\mathcal{G}_{x'x'}^{\mathrm{MM}}(0) = \mathcal{G}_{xx}^{\mathrm{MM}}(0)$ and $\mathcal{G}_{zx'}^{\mathrm{EM}}(\mathbf{R}_{ij}) = \left[ \mathcal{G}_{zx}^{\mathrm{EM}}(\mathbf{R}_{ij}) - \mathcal{G}_{zy}^{\mathrm{EM}}(\mathbf{R}_{ij}) \right] / \sqrt{2}$. Noticing that the monopartite arrays do not support a magnetic lattice resonance allows us to approximate $\mathcal{A}_{j,x'x'}^{\mathrm{MM}}$ as $\alpha_{j,x'}^{\mathrm{MM}}$, which leads to the expressions given in the main text.

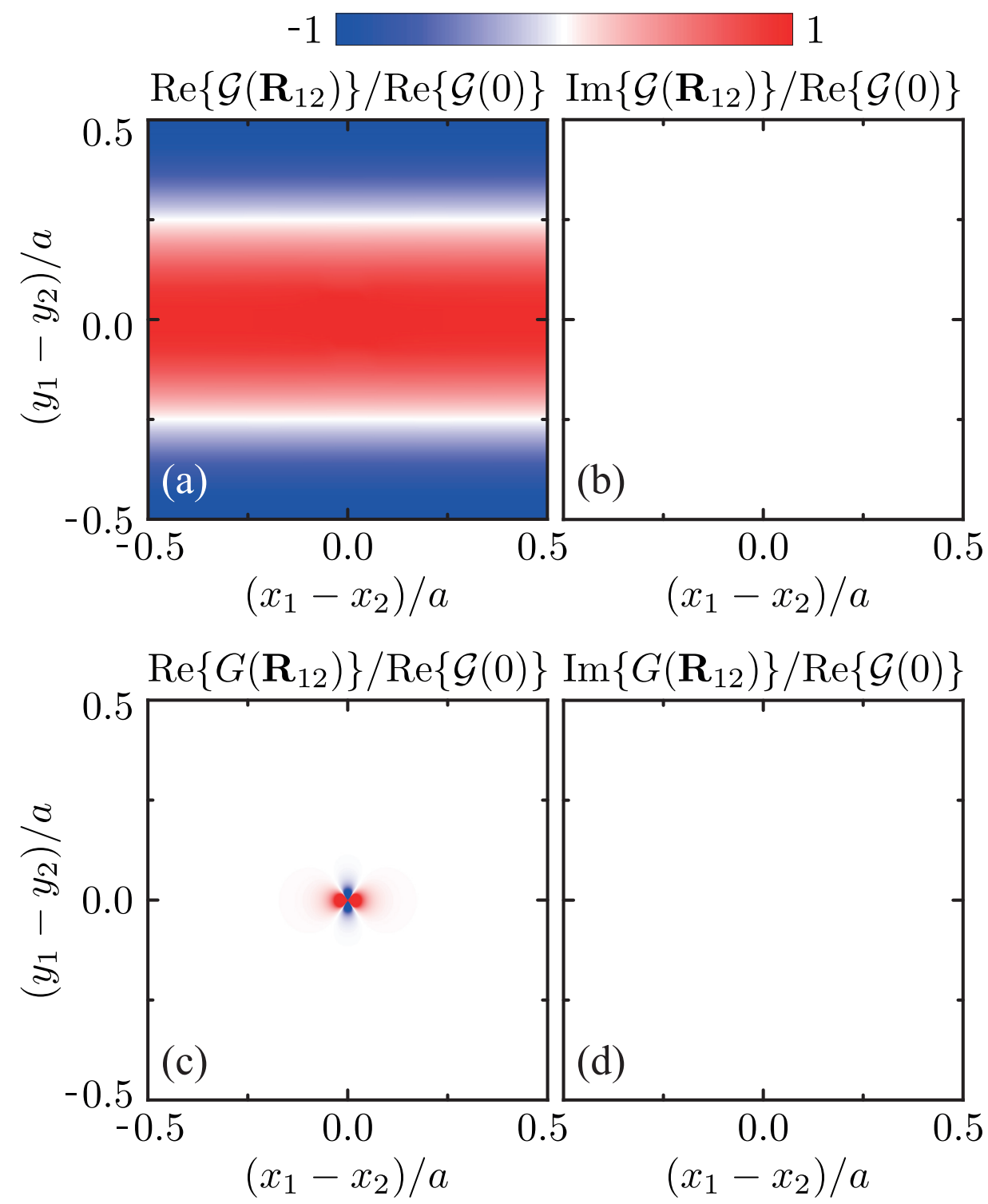


Figure S2: (a,b) Real (a) and imaginary (b) parts of the $xx$ component of the lattice sum, $\mathrm{Re}\{\mathcal{G}(\mathbf{R}_{12})\}$, calculated at $\mathbf{k}_\parallel = 0$ for two square arrays of period $a$ embedded in a homogeneous medium with refractive index $n$, plotted as a function of their relative displacement. (c,d) Real (c) and imaginary (d) parts of the $xx$ component of the Green's tensor, $\mathrm{Re}\{G(\mathbf{R}_{12})\}$, of a homogeneous medium with refractive index $n$, plotted as a function of $\mathbf{R}_{12}$. We perform all calculations at $\lambda = (1 + 10^{-6})an$, corresponding to a wavelength marginally exceeding the first Rayleigh anomaly of the arrays, and normalize the results to $\mathrm{Re}\{\mathcal{G}(0)\}$.

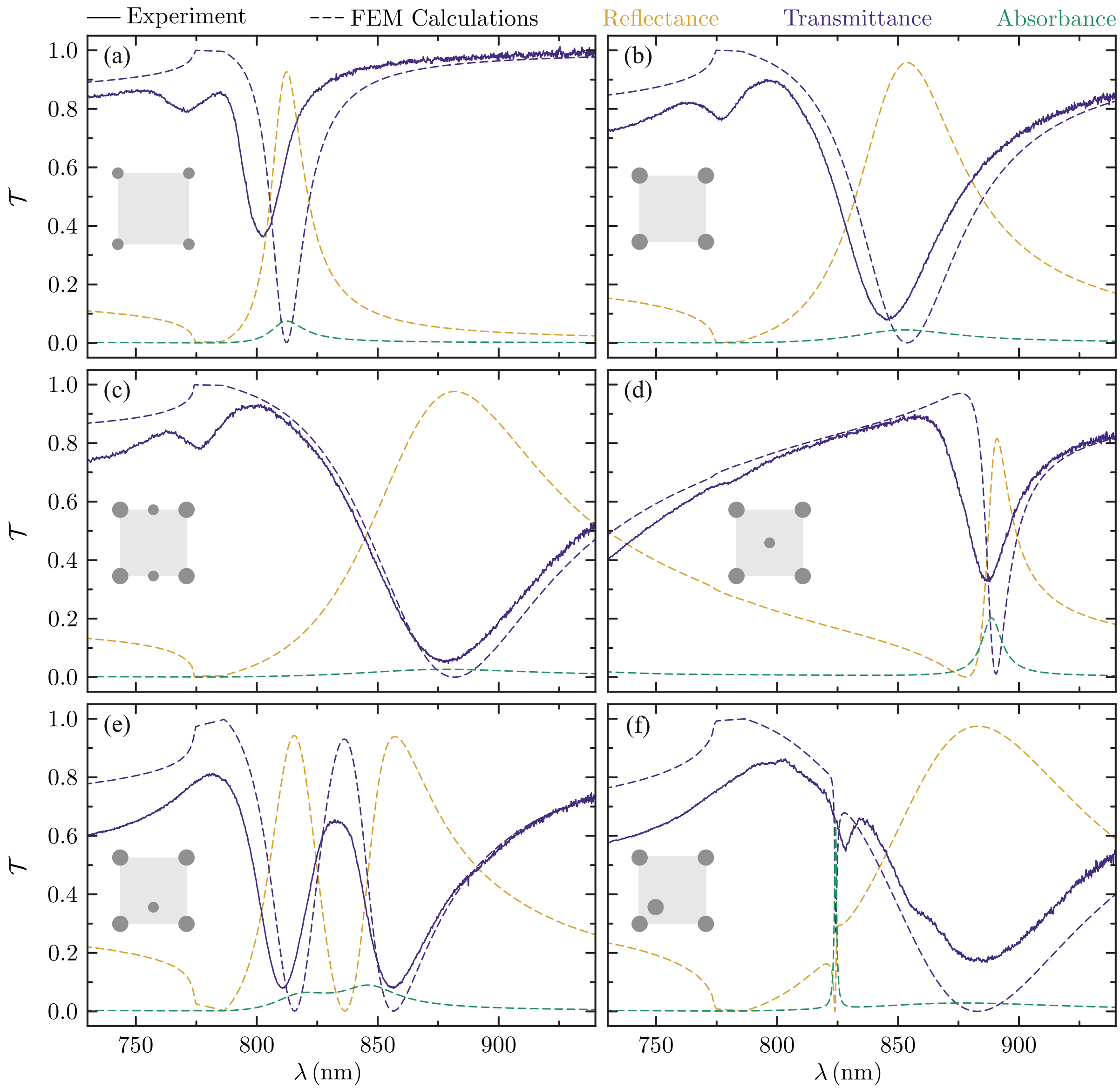


Figure S3: (a-f) Reflectance (orange curves), transmittance (purple curves), and absorbance (green curves) spectra for the periodic arrays of nanodisks discussed in the main text. Panels (a) and (b) correspond to monopartite arrays with nanodisk diameters $D = 140\,\text{nm}$ (a) and $D = 180\,\text{nm}$ (b). Panels (c-e) correspond to the superradiant (c), subradiant (d), and noninteracting (e) bipartite array configurations obtained by combining the monopartite arrays of panels (a) and (b). Panel (f) corresponds to the out-of-plane configuration built from two monopartite arrays of nanodisks with $D = 160\,\text{nm}$. In all cases, solid curves represent experimental measurements (only transmittance), while dashed curves correspond to FEM calculations. Each panel also includes a schematic of the nanodisk arrangement within the unit cell.

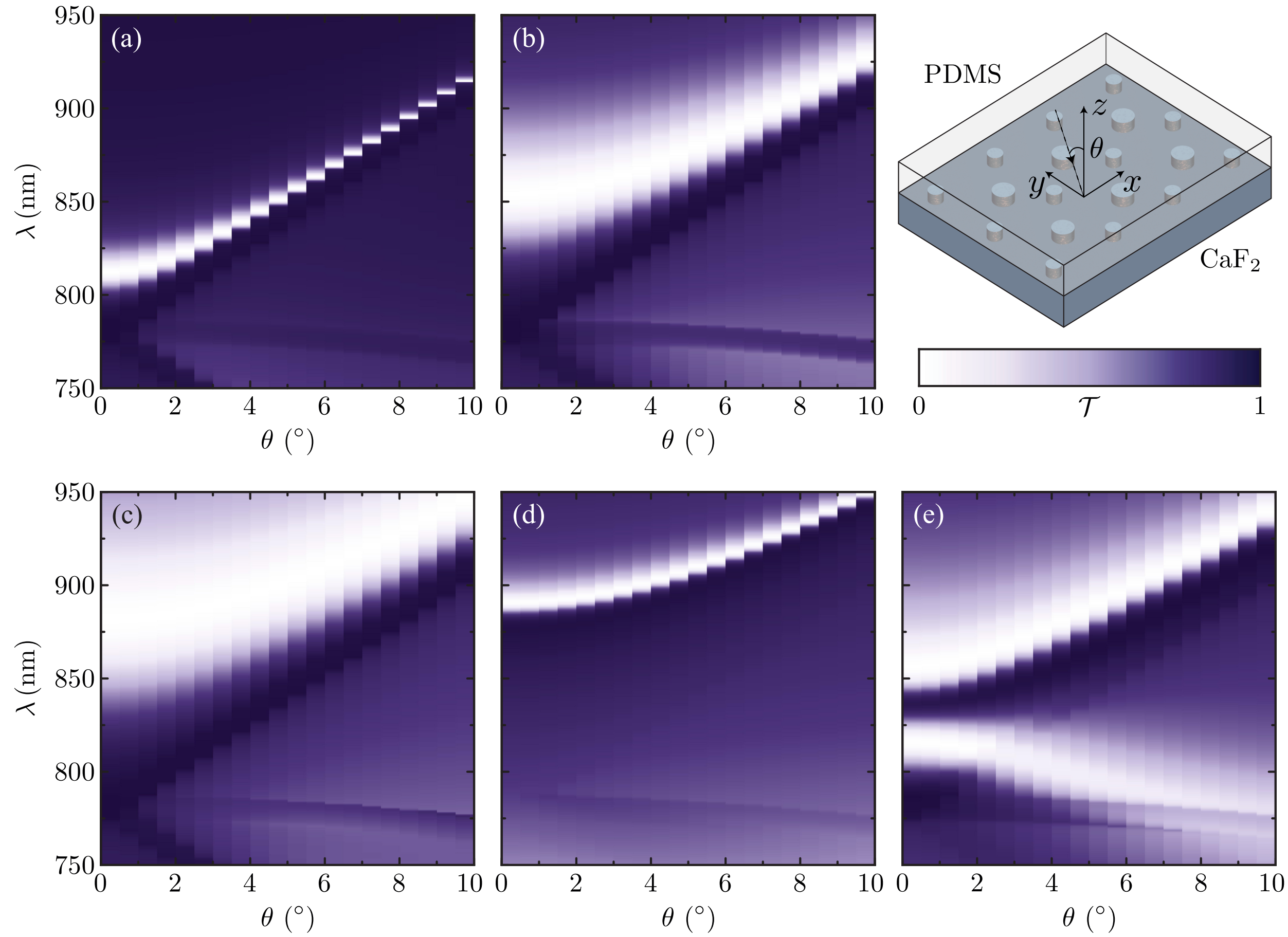


Figure S4: Transmittance maps calculated using the FEM for the periodic arrays of silver nanodisks supporting in-plane lattice resonances discussed in the main text, under oblique-incidence excitation. Panels (a) and (b) correspond to monopartite arrays with nanodisk diameters $D = 140\,\mathrm{nm}$ (a) and $D = 180\,\mathrm{nm}$ (b), while panels (c-e) correspond to the superradiant (c), subradiant (d), and noninteracting (e) bipartite configurations obtained by combining the monopartite arrays of panels (a) and (b). We plot the transmittance as a function of wavelength and angle of incidence $\theta$ inside PDMS, varied from $0°$ to $10°$ in steps of $0.5°$. The incident electric field is polarized along the $x$ axis, and the wavevector lies in the $yz$ plane, with $\theta$ measured from the $z$ axis, as illustrated in the sketch. The color scale is inverted, with higher (lower) values of transmittance shown in darker (lighter) tones, so that the lattice resonance appears as a bright feature in each panel.

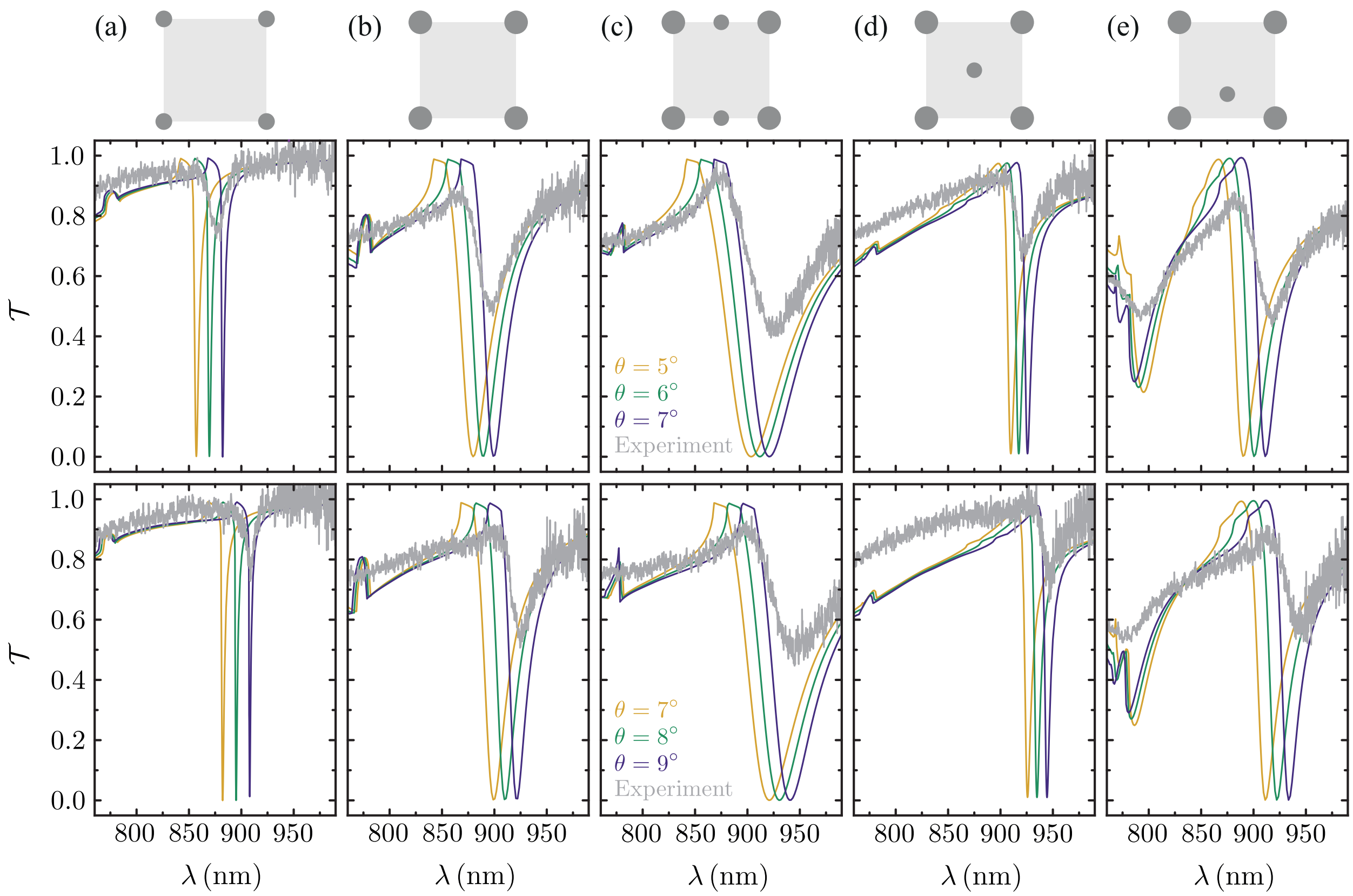


Figure S5: Transmittance spectra for the different arrays supporting an in-plane lattice resonance discussed in the main text, under oblique-incidence excitation. Panels (a-b) correspond to monopartite arrays with nanodisk diameters $D = 140\,\mathrm{nm}$ (a) and $D = 180\,\mathrm{nm}$ (b), while panels (c-e) show the superradiant (c), subradiant (d), and noninteracting (e) bipartite configurations. In all cases, gray curves represent experimental measurements at angles of incidence $\theta = (6 \pm 2)^\circ$ (top row) and $\theta = (8 \pm 2)^\circ$ (bottom row), with $\theta$ defined inside the PDMS layer. Colored curves correspond to FEM calculations at different angles of incidence, as indicated in the legends. We observe slight offset between the experimental measurements and FEM calculations, consistent with the experimental uncertainty in $\theta$.

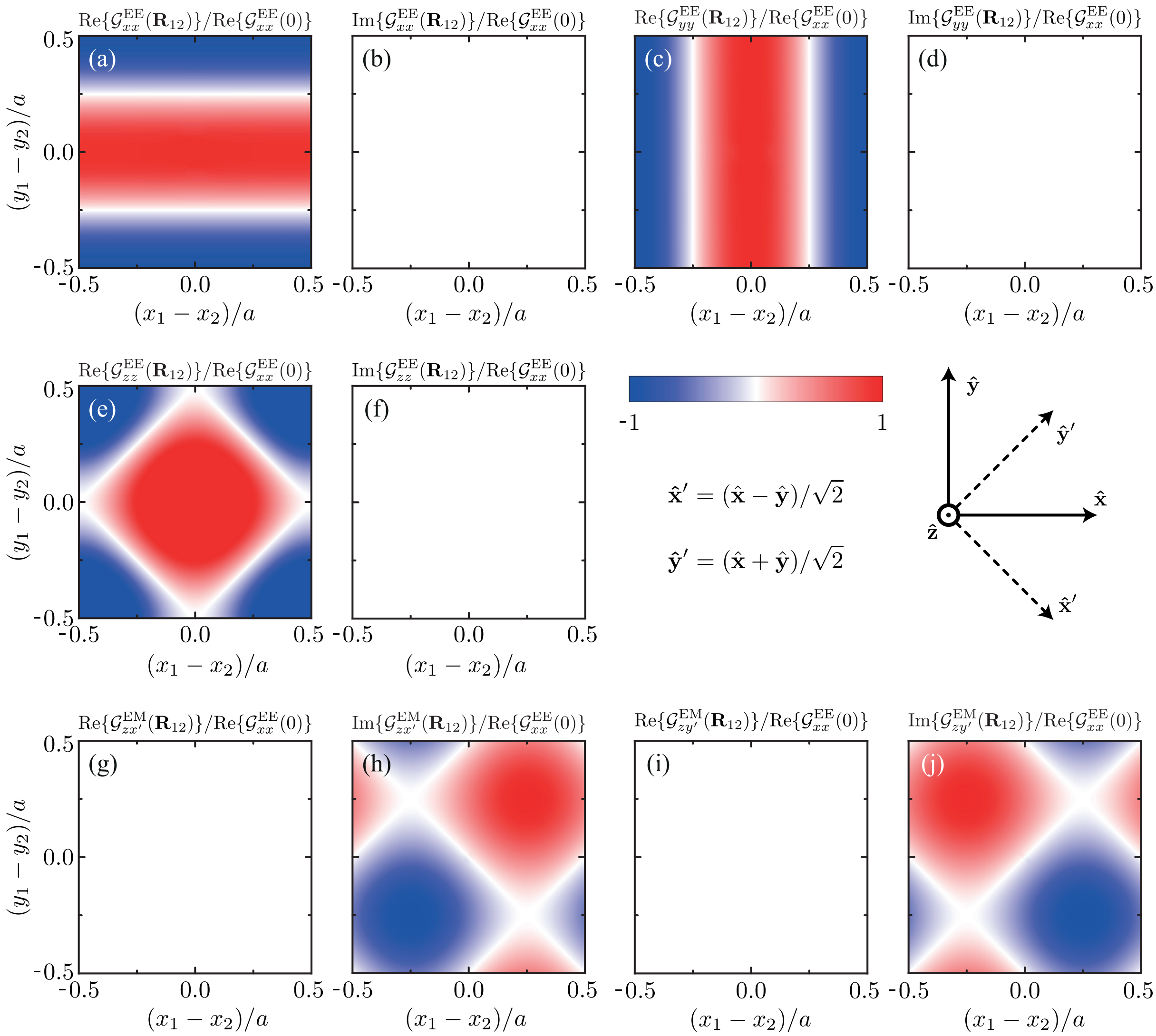


Figure S6: (a-j) Real and imaginary parts of the relevant lattice sum terms, $\boldsymbol{\mathcal{G}}(\mathbf{R}_{12})$, calculated at $\mathbf{k}_{\parallel} = 0$ for two square arrays of period $a$ embedded in a homogeneous medium with refractive index $n$, plotted as a function of their relative displacement. Panels (a-f) correspond to the $xx$ component (a,b), $yy$ component (c,d), and $zz$ component (e,f) of the electric-electric terms, $\boldsymbol{\mathcal{G}}^{\mathrm{EE}}(\mathbf{R}_{12})$. Panels (g-j) correspond to the $zx'$ component (g,h) and the $zy'$ component (i,j) of the electric-magnetic terms, $\boldsymbol{\mathcal{G}}^{\mathrm{EM}}(\mathbf{R}_{12})$. We perform all calculations at $\lambda = (1 + 10^{-6})an$, corresponding to a wavelength marginally exceeding the first Rayleigh anomaly of the array, and normalize the results to $\mathrm{Re}\{\mathcal{G}(0)\}$. The inset in the middle row shows the coordinate axes $xyz$ and $x'y'z$, together with the relation between their unit vectors.

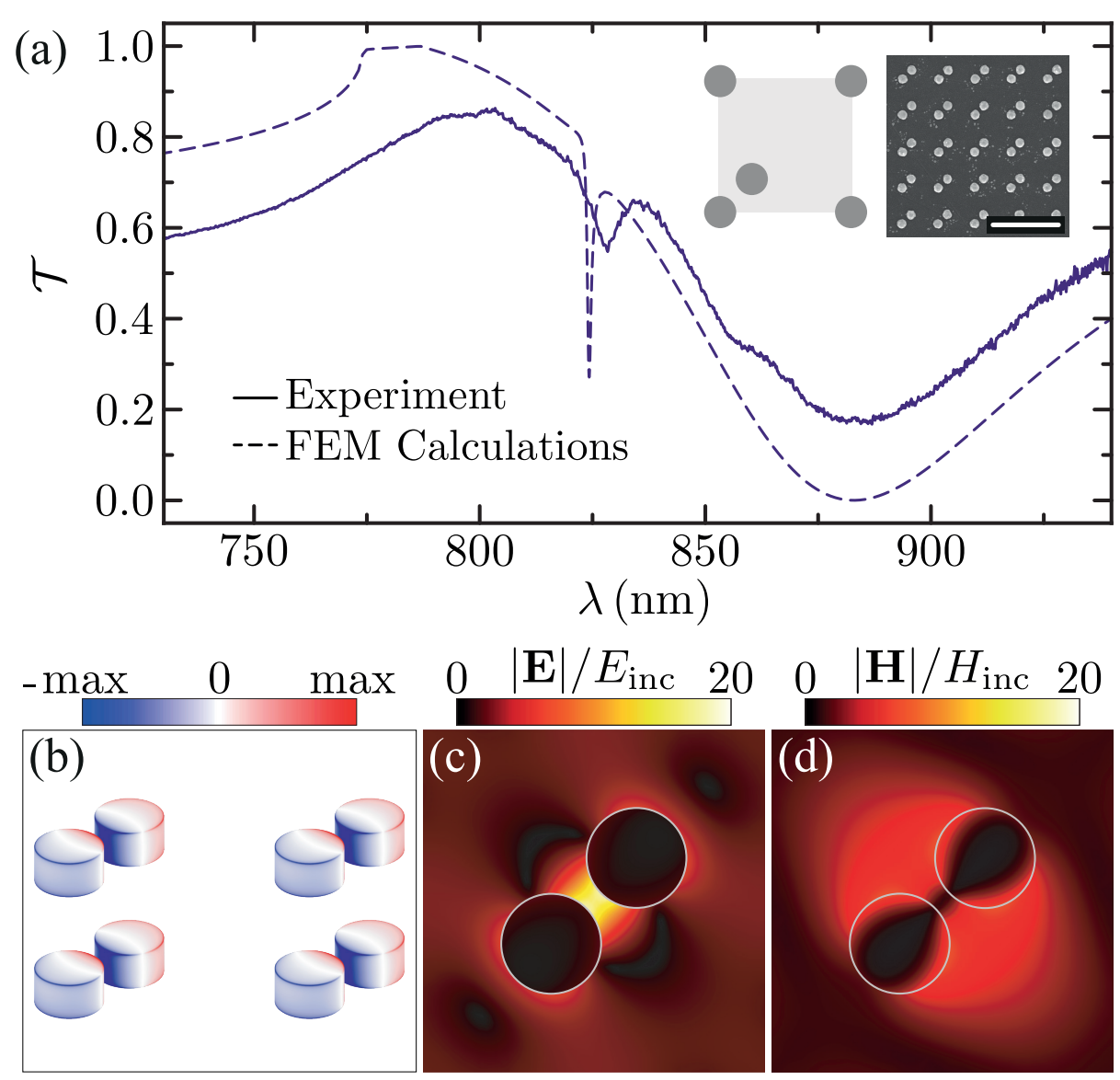


Figure S7: (a) Transmittance spectra for the bipartite array of nanodisks in the out-of-plane configuration. The two nanodisks in the unit cell are identical, with diameter $D = 160\,\text{nm}$. Solid and dashed curves represent experimental measurements and FEM calculations, respectively. The insets show a schematics of the nanodisk arrangement within the unit cell, along with an SEM image of the fabricated array (the scale bar represents $1\,\mu$m). (b) Charge density on the surface of the nanodisks. (c,d) Electric (c) and magnetic (d) field enhancements across the unit cell, evaluated at the plane $z = h/2$. Both the charges and field enhancements are calculated at the wavelength of the in-plane lattice resonance ($\lambda = 883.2\,\text{nm}$).

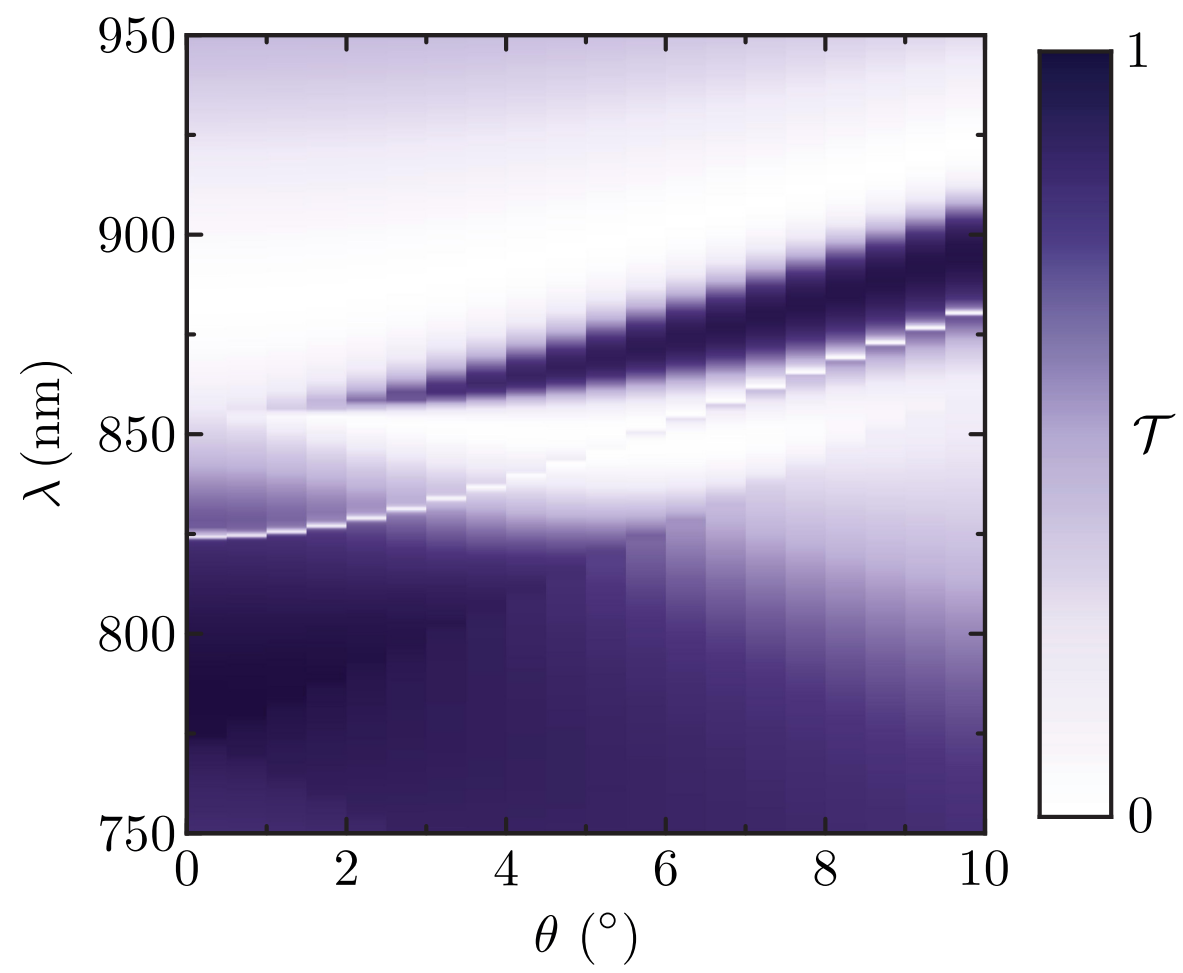


Figure S8: Transmittance map calculated using the FEM for the bipartite array of nanodisks in the out-of-plane configuration, under oblique-incidence excitation. The two nanodisks in the unit cell are identical, with diameter $D = 160\,\mathrm{nm}$. We plot the transmittance as a function of wavelength and angle of incidence $\theta$ inside PDMS, varied from 0° to 10° in steps of 0.5°. The incident electric field is polarized along the $y'$ axis, and the wavevector lies in the $x'z$ plane, with $\theta$ measured from the $z$ axis. The color scale is inverted, with higher (lower) values of transmittance shown in darker (lighter) tones, so that the lattice resonance appears as a bright feature in each panel.
